# Quantitative comparison of two-dimensional and three-dimensional strain measurement using MRI feature tracking in repair Fontan patients and normal child volunteers

**DOI:** 10.1186/s12880-020-0413-6

**Published:** 2020-01-28

**Authors:** Liwei Hu, Qian Wang, Barton P. Gregory, Rong Zhen Ouyang, Aimin Sun, Chen Guo, Tongtong Han, Yumin Zhong

**Affiliations:** 1 Diagnostic Imaging Center of Shanghai Children’s Medical Center affiliated with Shanghai Jiao Tong University School of Medicine, 1678 Dong Fang Road, Shanghai, 200127 People’s Republic of China; 2 Radiology, University of Wisconsin-Madison, Madison, WI USA; 3 Pediatrics, University of Wisconsin-Madison, Madison, WI USA; 4 Circle Cardiovascular Imaging, 250, 815 8th Ave SW, Calgary, Canada

**Keywords:** Strain, Feature tracking, Fontan operation, Pediatric

## Abstract

**Background:**

The accuracy of 2D and 3D strain analyses was evaluated by comparing strain and cardiac function parameters in Fontan repair patients and normal child volunteers.

**Methods:**

We retrospectively enrolled 32 patients with Fontan circulation and 32 child volunteers who had undergone clinical cardiac magnetic resonance (CMR) assessment of the dominant ventricle with a 1.5-Tesla MRI scanner. Global and regional strain (2D and 3D) of the dominant ventricle in both groups was assessed using CMR feature-tracking. Correlations between cardiac function and strain data were assessed using Pearson’s correlation coefficient values. The intraclass correlation coefficient (ICC) and coefficient of variation (CoV) were determined to evaluate repeatability and agreement.

**Results:**

The 2D GLS showed significant differences between the Fontan repair patients and volunteers (− 16.49 ± 5.00 vs. -19.49 ± 2.03; *p* = 0.002). The 2D GRS and 2D GCS showed no significant differences between two groups. 2D GRS: 38.96 ± 14.48 vs. 37.46 ± 7.77; 2D GCS: − 17.64 ± 5.00 vs. -16.89 ± 2.96, respectively; *p* > 0.05). The 3D global radial strain (GRS), global circumferential strain (GCS), and global longitudinal strain (GLS) showed significant differences between the Fontan repair patients and volunteers (3D GRS: 36.35 ± 16.72 vs. 44.96 ± 9.98; 3D GLS: − 8.86 ± 6.84 vs. -13.67 ± 2.44; 3D GCS: − 13.70 ± 7.84 vs. -18.01 ± 1.78; *p* < 0.05, respectively). The ejection fraction (EF) and 3D GCS were significantly associated (*r* = − 0.491, *p* = 0.004). The 3D GCS showed correlations with the indexed end-diastolic volume (EDV) (*r* = 0.523, *p* = 0.002) and indexed end-systolic volume (ESV) (*r* = 0.602, *p* < 0.001). 3D strain showed good reproducibility, with GCS showing the best inter-observer agreement (ICC = 0.87 and CoV = 5.15), followed by GLS (ICC = 0.84 and CoV = 5.36).

**Conclusions:**

3D GCS is feasible, highly reproducible, and strongly correlated with conventional cardiac function measures. 3D GCS assessments may be useful for monitoring abnormal myocardial motion in patients with Fontan circulation.

## Background

Functional single ventricle (FSV) is a type of severe congenital heart disease (CHD) [1]. The first palliation surgery for FSV was described by Fontan and Baudet [2] and performed in 1971 to treat tricuspid atresia. In patients who have undergone this Fontan repair procedure, a single ventricle provides blood flow in series to the pulmonary and systemic circulation. Young survivors with Fontan circulation commonly develop ventricular dysfunction, which has been identified as a risk factor for mortality [3, 4]. Moreover, long-term follow-up studies over the last 20 years have used echocardiography (echo) to assess ventricular function after successful Fontan surgery in childhood and shown significant differences in regional deformation and ejection fraction [5–7].

Cardiac magnetic resonance (CMR) has recently become the gold standard for measurement of ventricular function and myocardial motion [8]. CMR feature tracking (FT) has been adapted and applied to the standard CMR sequence (balanced steady-state free precession, b-SSFP) without additional sequences, unlike in myocardial tagging or displacement encoding with stimulated echoes (DENSE) imaging [9]. Strain assessment by both echo and CMR shows high intra-modality and modest inter-modality reproducibility [10]. At present, our study found that two-dimensional (2D) strain analysis is considered a suitable tool to detect early abnormalities of the ventricular myocardium [11]. However, ventricular function assessment is challenging in post-Fontan FSV patients due to the complex ventricular geometry. Some studies have indicated that three-dimensional (3D) strain analysis may overcome such geometry-dependent limitations of 2D strain analysis by referencing the intrinsic directions of deformation [12, 13]. To our knowledge, there are no previous studies assessing three-dimensional strain analysis of SSFP cine images in Fontan patients and normal volunteers [14]. Thus, the aim of this study is to explore the feasibility of 3D strain analysis with 2D cine CMR images that may be clinically useful in the assessment of post Fontan patients.

## Methods

### Study population

**Fig. 1:**
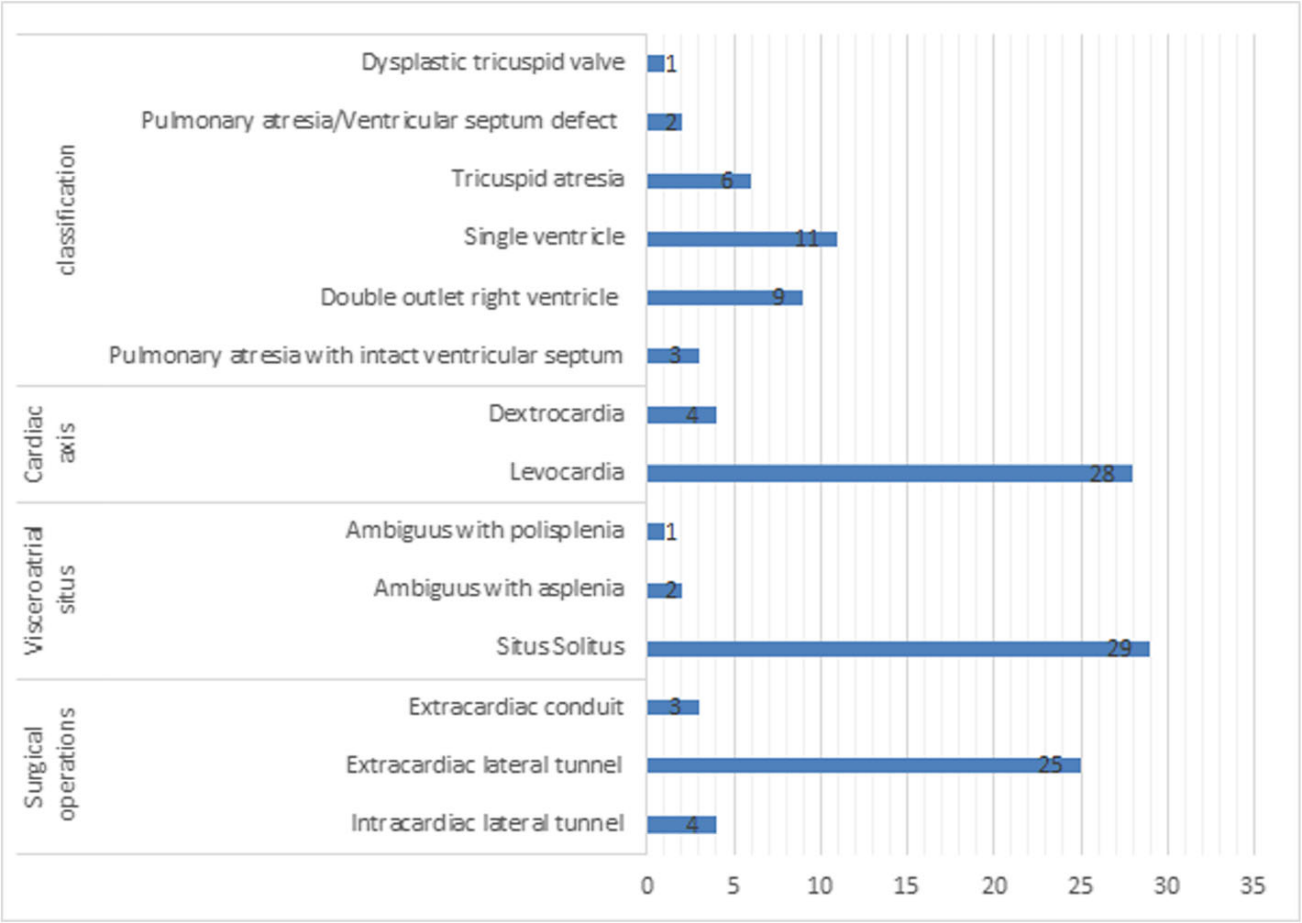
The clinical and diagnostic information in repaired Fontan patients

Our study was approved by the ethics committee of our hospital (Institutional Review Board of Shanghai Children’s Medical Center) and was conducted in accordance with the Declaration of Helsinki. Thirty-two Fontan repair patients (male/female, 21/11) were retrospectively enrolled from June 2015 through August 2017. Databases were reviewed to identify patients who had undergone the Fontan procedure in infancy or early childhood (Fig. 1). Then, patients who met the following criteria were retrospectively enrolled: 1) previously undergone the Fontan operation, and 2) no history of intervening surgery or catheter procedures for at least 1 year before CMR examination. The exclusion criteria were as follows: 1) presence of other diseases that could influence cardiac function in children (e.g., pulmonary hypertension, arrhythmia, valvular stenosis, or moderate to severe valvular regurgitation); 2) presence of serious liver, kidney, or lung dysfunction; and 3) inadequate image quality for analysis. Furthermore, informed consent was obtained from the parents of all the participating children. The control group consisted of 32 gender-matched healthy children with no history of cardiac disease and with LVEF ≥55% confirmed by echocardiography. All participants were screened using a health questionnaire and by elicitation of detailed family medical history. None of the subjects had sedation before MRI and echocardiography examination.

### Cardiac magnetic resonance

All images were acquired using a Philips 1.5 Tesla MR Achieva system (Philips Healthcare, Best, Netherlands) with an 8-element cardiac phased array coil. Ventricular function and strain were assessed in the serial contiguous cardiac short-axis and long-axis planes (in 2- and 4-chamber views) by using a standard balanced steady-state free precession (b-SSFP) cine sequence. The parameters were as follows: repetition time (ms)/echo time (ms), 3.5/1.7; voxel size, 1.3 × 1.6 × 6–8 mm; flip angle, 60°; SENSE acceleration factor, 2.0; bandwidth, 130.8 kHz/pixel; and 20–28 phases per cardiac cycle.

### Functional analysis

Cine image analysis was performed using Circle Cardiovascular Imaging software (cvi42® version 5.6.1, Circle Cardiovascular Imaging, Canada) by two individuals with more than 10 years of experience in cardiac functional analysis who were blinded to patient demographic data. The endocardial and epicardial borders of the dominant ventricle were manually traced on short-axis cine images at end-systole and end-diastole. Papillary muscles, trabeculae, and the rudimentary ventricle [15] were not included in FSV volumes and were excluded from the dominant ventricle mass. The ventricle end-diastolic volume (EDV), end-systolic volume (ESV), and mass were obtained from short-axis stacks. Stroke volume (SV) and ejection fraction (EF) were calculated automatically. Height and weight were measured, and body surface area (BSA) was calculated. Ventricular volume and mass were indexed to the BSA (Table 1).

**Table 1:** CMR measurements for the patient and control groups (mean ± SD)

Variables	Patient group (*n* = 32)	Control group (*n* = 32)	*p* value
Age at CMR (years)	9.5 ± 3.41	13.12 ± 2.87	**< 0.001**
Males (%)	21 (65%)	21 (65%)	
Post-surgery follow-up time (years)	5.06 ± 2.24	–	
Heart rate (beats/min)	84 ± 21	72 ± 13	0.025
Body surface area (m^2^)	1.04 ± 0.29	1.48 ± 0.23	**< 0.001**
EDV_i_ (ml/m^2^)	76.21 ± 44.75	75.15 ± 10.98	0.903
ESV_i_ (ml/m^2^)	34.40 ± 26.09	28.92 ± 6.46	0.536
Stroke volume_i_ (ml)	43.25 ± 21.49	46.22 ± 7.84	0.210
Ejection fraction	58.85 ± 10.67	61.56 ± 6.03	0.138
Ventricle mass_i_ (g)	47.77 ± 28.81	90.40 ± 25.83	**0.003**
SBP (mm Hg)	112 ± 8	115 ± 7	
DBP (mm Hg)	67 ± 6	67 ± 6	

There were statistical differences in bold representation

*i* indexed

### Strain analysis

**Fig. 2:**
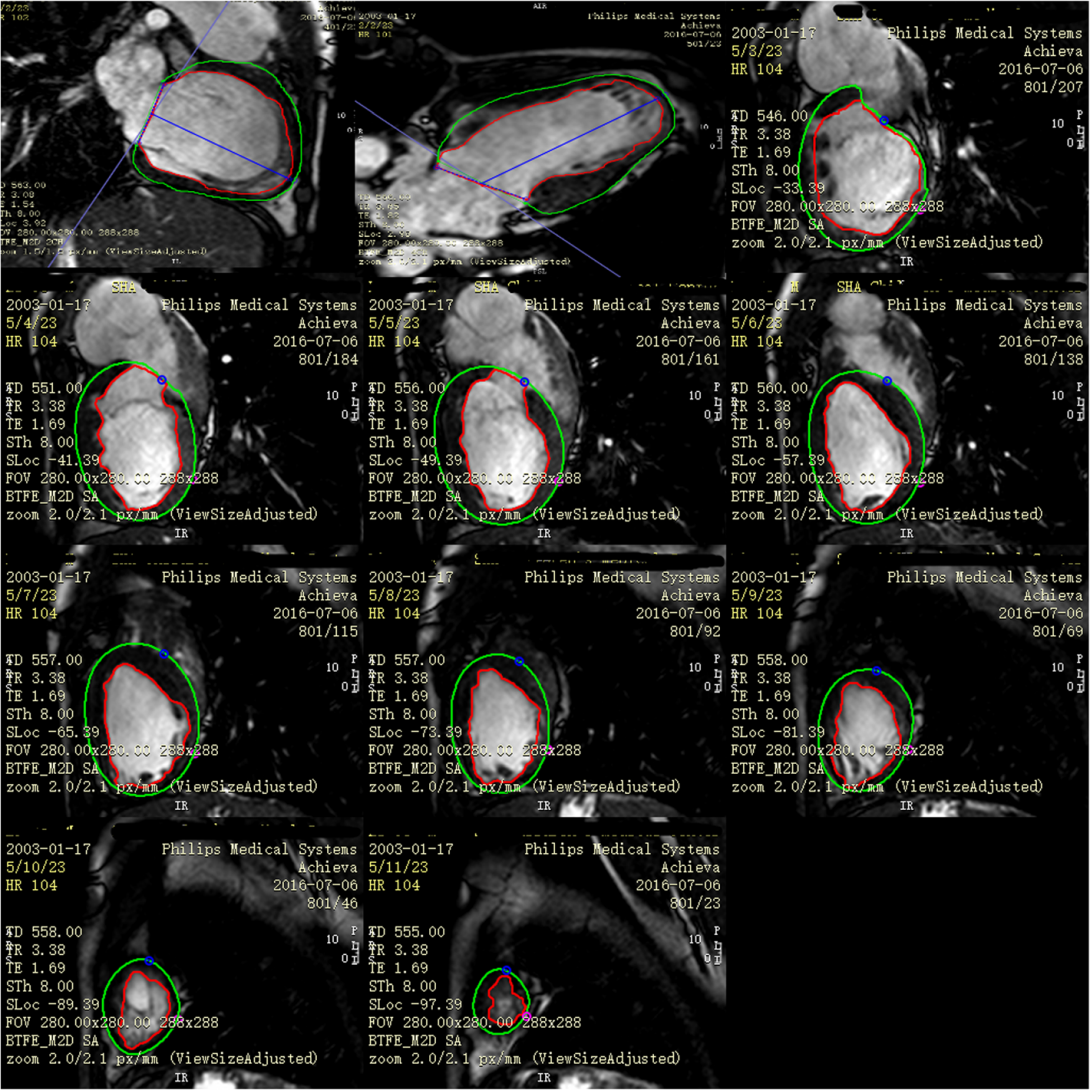
2D global longitudinal strain and global circumferential strain and global radial strain were taken from the short-axis and four-chamber planes from basal to apical slices at end-diastole in the post-Fontan patient case. (endocardial: red line; epicardial borders: green circle)

**Fig. 3:**
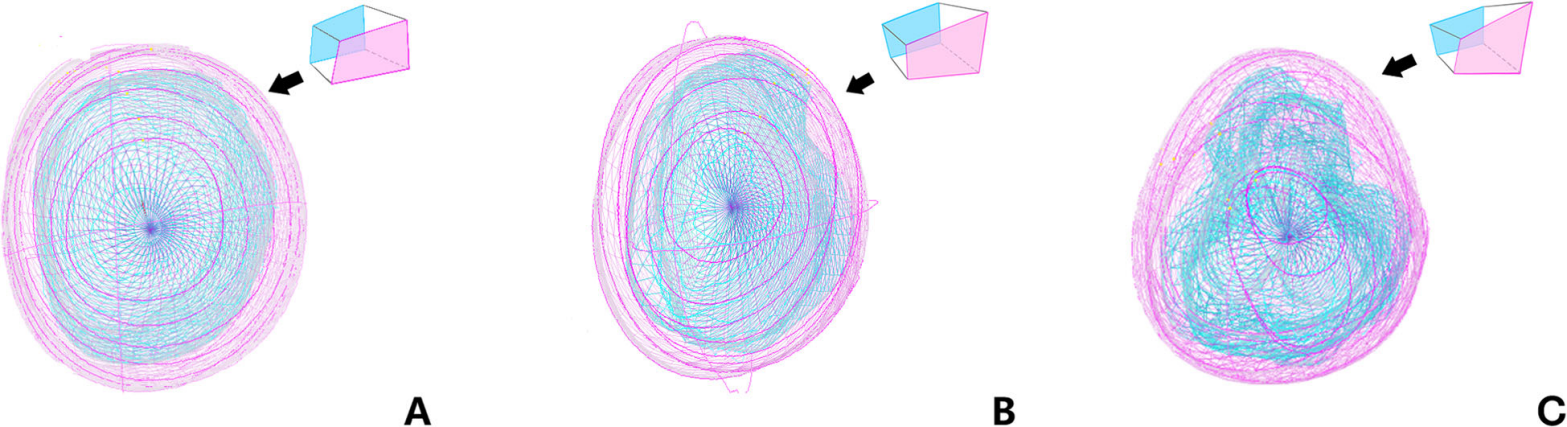
**a** Show 3D mesh model of volunteer; **b** show 3D mesh map of single left ventricular morphology; **c** show 3D mesh map of single right ventricular morphology. This mesh model was corresponding points (nodes) from respective endocardial and epicardial surfaces coupled to obtain a hexahedral 3D mesh based on a deformable model. Deformations for each hexahedral element were then calculated using a Lagrangian strain definition

The FT module was used to perform strain analysis in the patient and control groups using cvi42 software. The semi-manually defined endocardial and epicardial borders’ contour at the end-diastole served as the starting point from which the software tracked displacement of spatial features in successive sequence images (Fig. 2). 2D global longitudinal strain (GLS) was measured from the two- and four-chamber planes of the dominant ventricle at end-diastole, and global circumferential strain (GCS) and global radial strain (GRS) were taken from the short-axis and four-chamber planes from the basal to the apical slices. In 3D strain analysis, a 3D deformable model of the myocardium is generated in the end-diastolic phase by interpolating the endo- and epicardial boundaries tracked by the 2D algorithm (Fig. 3).

### Statistical analysis

All data were presented as means ± standard deviations or numbers (percentage) and ranges. Comparison of cardiac function and strain data between Fontan repair patients and volunteers was performed using the *t* test for normally distributed variables and the Wilcoxon test for non-normally distributed variables. Correlations were assessed with Pearson’s correlation coefficient between cardiac function and strain data. Bland Altman analysis, intraclass correlation coefficient (ICC) and coefficient of variation (CoV) were obtained to evaluate the repeatability and agreement. A *p*-value of < 0.05 was considered statistically significant. Statistical analyses were performed with SPSS version 22.0 software package (IBM Corp., Armonk, NY, USA).

### Reproducibility studies

A single observer reviewed all CMR-FT studies and performed the image analyses. To assess intra-observer variability, the same investigator re-analyzed 10 random studies continuously 2 weeks after the first analysis. To evaluate inter-observer reproducibility, the same cases were then evaluated by a second observer who was blinded to the results recorded by the first observer. Intra-observer and inter-observer agreement for 2D/3D global radial, circumferential and longitudinal strain were measured using Bland Altman method by CMR with 10 random studies.

## Results

### Subgroup functional analysis

Cardiac function parameters of the 32 study subjects and 32 control children are presented in Table 1. The FSV showed a left ventricular morphology in 21 cases and a right ventricular morphology in 11 cases. The two groups showed significant differences in mean ages (9.5 ± 3.41 vs. 13.12 ± 2.87 years), mean heart rates (84 ± 21 vs. 72 ± 13 beats/min), mean BSAs (1.04 ± 0.29 vs. 1.48 ± 0.23 m^2^), and ventricle mass (47.77 ± 28.81 vs. 90.40 ± 25.83 g). However, there were no statistically significant CMR volumetric differences between the groups (*p* > 0.05 for all; Table 1).

### Strain analysis

The mean GRS, GCS, and GLS (2D and 3D) values for the two groups are presented in Table 2. The 2D GLS showed significant differences between the Fontan repair patients and volunteers (− 16.49 ± 5.00 vs. -19.49 ± 2.03; *p* = 0.002; Table 2). The 2D GRS and 2D GCS showed no significant differences between two groups. 2D GRS: 38.96 ± 14.48 vs. 37.46 ± 7.77; 2D GCS: − 17.64 ± 5.00 vs. -16.89 ± 2.96, respectively; *p* > 0.05; Table 2). The 3D GRS, 3D GCS, and 3D GLS all showed significant differences between the Fontan repair patients and volunteers (3D GRS: 36.35 ± 16.72 vs. 44.96 ± 9.98; 3D GLS: − 8.86 ± 6.84 vs. -13.67 ± 2.44; 3D GCS: − 13.70 ± 7.84 vs. -18.01 ± 1.78, respectively; *p* < 0.05; Table 2).

**Table 2:** 2D and 3D global radial, circumferential, and longitudinal strain in the patient and control groups

Variables	Patient group (*n* = 32)	Control group (*n* = 32)	*p* value
Radial			
2D strain	38.96 ± 14.48	37.46 ± 7.77	0.567
3D strain	36.35 ± 16.72	44.96 ± 9.98	**0.010**
Longitudinal			
2D strain	−16.49 ± 5.00	−19.49 ± 2.03	**0.002**
3D strain	−8.86 ± 6.84	−13.67 ± 2.44	**< 0.001**
Circumferential			
2D strain	−17.64 ± 5.00	− 16.89 ± 2.96	0.440
3D strain	−13.70 ± 7.84	−18.01 ± 1.78	**0.003**

There were statistical differences in bold representation

The 2D GCS were highly correlated with cardiac function in Fontan repair patients (EF: *r* = − 0.612, *p* < 0.001, EDV_i_: *r* = 0.471, *p* = 0.006, ESV_i_: *r* = 0.556, *p* = 0.001, Table 3) respectively. The 2D GRS have good correlation with cardiac function, (EF: *r* = 0.641, *p* < 0.001, EDV_i_: *r* = − 0.455, *p* = 0.008, ESV_i_: *r* = − 0.533, *p* = 0.002, Table 3) respectively. We also found a significant association between EF and 3D GCS (*r* = − 0.491, *p* = 0.004, Table 3). The indexed EDV and ESV correlated with 3D GCS (*r* = 0.523, *p* = 0.002, *r* = 0.602, *p* < 0.001, Table 3), respectively. The mean regional circumferential strain (2D and 3D) values were compared in the Fontan repair patients. At the base, the 3D circumferential strain was higher than the 2D circumferential strain (− 16.39 ± 5.63 vs. -12.67 ± 5.16, *p* = 0.001, Table 4). In the midmyocardium, 2D circumferential strain was not significantly different from 3D circumferential strain (− 19.32 ± 5.00 vs. -17.86 ± 5.00, *p* = 0.06, Table 4). At the apex, the 3D circumferential strain was significantly lower than the 2D circumferential strain (− 16.38 ± 4.66 vs. -22.00 ± 5.29, *p* < 0.001, Table 4).

**Table 3:** Correlation between cardiac function and global strain in the patient group

Cardiac function	Strain parameter	*R*	*P*
EDV_i_ (ml/m^2^)	2D GLS	0.312	0.082
	2D GCS	0.471	**0.006**
	2D GRS	−0.455	**0.008**
	3D GLS	0.103	0.572
	3D GCS	0.523	**0.002**
	3D GRS	−0.133	0.467
ESV_i_ (ml/m^2^)	2D GLS	0.455	0.008
	2D GCS	0.556	**0.001**
	2D GRS	−0.533	**0.002**
	3D GLS	0.212	0.245
	3D GCS	0.602	**< 0.001**
	3D GRS	−0.200	0.272
EF(%)	2D GLS	−0.539	0.001
	2D GCS	−0.612	**< 0.001**
	2D GRS	0.641	**< 0.001**
	3D GLS	−0.478	0.005
	3D GCS	−0.491	**0.004**
	3D GRS	0.347	0.05

There were statistical differences in bold representation

*i* indexed

**Table 4:** Comparison of 2D and 3D regional strain in the Fontan repair groups

(*n* = 32)	2D strain	3D strain	*p* value
Circumferential			
Basal	−12.67 ± 5.16	−16.39 ± 5.63	**0.001**
Mid	−19.32 ± 5.00	−17.86 ± 5.00	0.06
Apical	−22.00 ± 5.29	−16.38 ± 4.66	**< 0.001**

There were statistical differences in bold representation

### Reproducibility studies

The 2D global strain assessments showed good intra- and inter-observer agreement, except the inter-observer agreement for 2D GRS. The 3D strain showed better reproducibility than did 2D strain assessments, with GCS showing the best inter-observer agreement (ICC, 0.87 and CoV = 5.15), followed by GLS (ICC 0.84 and CoV = 5.36) and GRS (ICC 0.80 and CoV = 10.29) (Table 5).

**Table 5:** Intra-observer and inter-observer variability of CMR for 2D and 3D global strain in randomly selected 10 cases from patients with Fontan repair and controls

Variables	Intra-observer (*n* = 10)		Inter-observer (*n* = 10)	
	ICC (%)	CoV	ICC (%)	CoV
2D GLS (%)	0.91 (0.84–0.94)	3.13	0.84 (0.76–0.89)	5.12
2D GCS (%)	0.86 (0.78–0.90)	4.92	0.82 (0.73–0.88)	5.73
2D GRS (%)	0.88 (0.80–0.93)	7.18	0.76 (0.62–0.85)	11.23
3D GLS (%)	0.83 (0.76–0.88)	3.62	0.84 (0.74–0.86)	5.36
3D GCS (%)	0.89 (0.83–0.92)	4.59	0.87 (0.79–0.94)	5.15
3D GRS (%)	0.81 (0.72–0.87)	8.17	0.80 (0.67–0.88)	10.29

## Discussion

The Fontan procedure is the final step in the staged palliative repair of FSV [16], and children who have undergone this procedure are at risk for clinical complications. Fontan repair patients show comparatively good preservation of ventricular function in the intermediate term; however, ventricular function deteriorates in most patients over time [17]. Ventricular dysfunction attributed to long-term volume and pressure overload could have been more prominent in the post-Fontan operation by average five-years follow-up.

CMR-FT is a non-invasive modality for evaluating ventricular function in Fontan repair patients. It has been used to analyze ventricular function in CHD, particularly in cases of coarctation of the aorta [18] and repaired transposition of the great arteries [19]. In the previous publications, Satriano A et al. mentioned that 3D principal strain analysis from routine 2D cine CMR imaging is clinical feasible, highly reproducible, and shows strong correlations with conventional measures of strain [20]. 3D strain clinical research had been reported in recent years [21]. Considering the difficulties in ventricular function assessment in Fontan FSV repair patients due to the complex ventricular geometry, 3D strain analysis by FT may be a more accurate tool than 2D strain analysis [12]. In a study of 100 healthy subjects. Liu et al. [22] showed that the 3D global strain values ranged from 22 to 73 for radial strain, − 9 to − 20 for circumferential strain, and − 13 to − 23 for longitudinal strain. In our study, the 32 control group volunteers showed strain values within the reference range. In another study by André et al. [23] that used parabolic regression equations, no sex-related differences were found in the assessed strain values in children and adolescents. Our study showed significant age differences between the patient group and control group. There were no statistically significant CMR volumetric differences between the groups despite this age discrepancy, which might suggest that FSV patients are already demonstrating ventricular dilatation. All three of the 3D GLS, GCS and GRS values in the Fontan repair patients was significantly lower than those in the child volunteers. In contrast, only the 2D global longitudinal strain showed significant differences, which has been consistently reported in previous studies. 2D GLS has been the most valuable clinical marker for the assessment of myocardial disease [24]. We found that the 3D global strain was lower than the 2D strain in the patient group. We propose the following explanation for this finding: In 2D CMR-FT, the out-of-plane motion of one segment exaggerates the perceived degree of muscle shortening, thereby resulting in overestimation of myocardial movement. 3D CMR-FT based on the 3D incompressible model-based algorithm can overcome the assumption of cylindrical chamber architecture and therefore produces lower values [20]. 3D strain assessments may be useful for monitoring abnormal regional myocardial motion in patients with Fontan circulation. In our study, 3D RCS decrease was marked in the basal and apical segments (*P* < 0.001). it could be related to abnormal cardiac looping, which leads to hearts that lack helical fiber patterns. However, this theory needs further study.

The 2D strain algorithm fits a 2D incompressible deformable cylindrical model of the myocardium to individual image slices over the entire cardiac cycle. This model is assumed to be completely determined by control points placed on the middle curve of the endo and epi-boundaries of the myocardial wall. The model is based on the feature-tracked boundaries and the incompressibility constraint of the model. In contrast, the 3D strain algorithm uses a 3D deformable model generated by interpolating the tracked boundaries from the 2D algorithm. The surface interpolation is performed using both long- and short-axis image information [25]. The removal of constraints through pre-determined geometry-dependent directions of deformation may therefore, provide a more accurate and reproducible measure of Fontan repair cases. On the basis of these principles, the 3D regional strain offers advantages in analyzing non-cylindrical structure models. In our study, patients with Fontan repair included 21 cases with left ventricular morphology and 11 cases with right ventricular morphology. In order to quantify strain must standardize anatomically the myocardial regions. However, it is challenging in patients with single RV. Due to the complex ventricular geometry, this result is reinforced by the regional deformation analysis of the different levels [20]. This difference between 2D and 3D regional strain needs to be investigated further in a patient group.

**Fig. 4:**
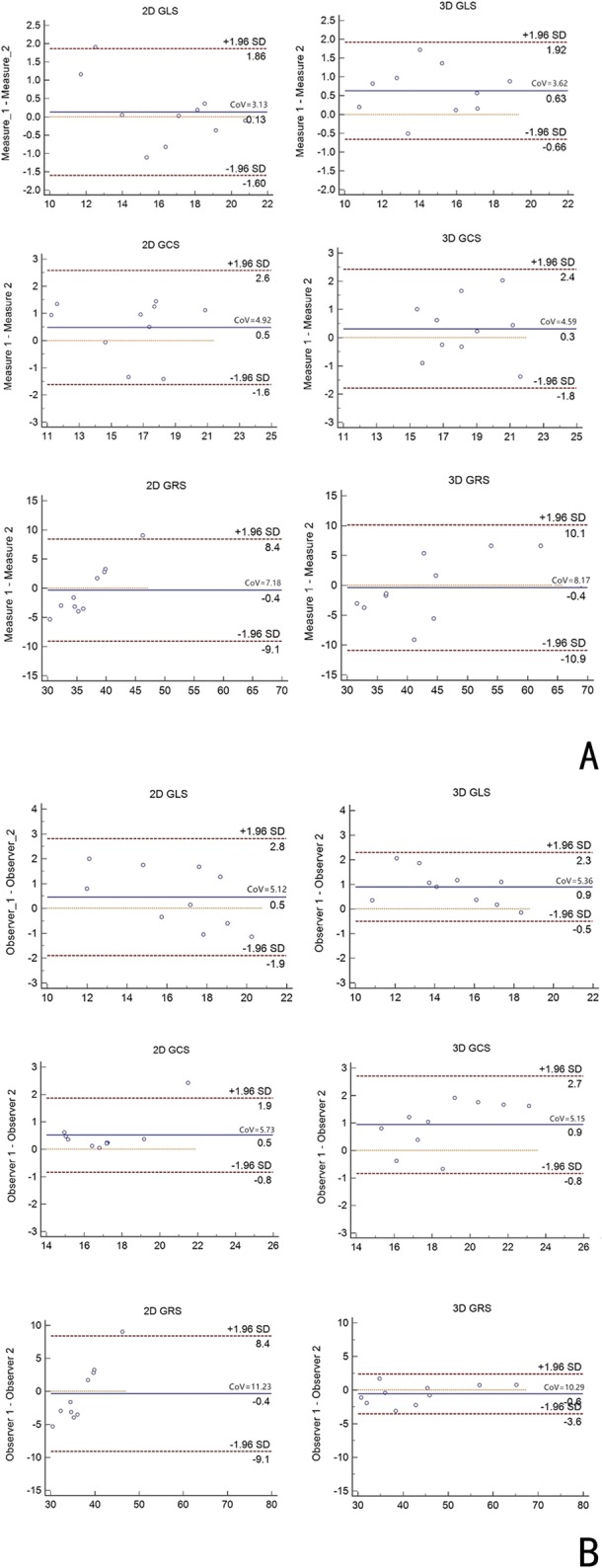
Intra-observer (**a**) and inter-observer agreement (**b**) for 2D/3D global radial, circumferential and longitudinal strain measurement by CMR with 10 random studies. Solid line indicated perfect agreement, dotted line indicated mean difference, dashed lines indicated 95% limits of agreement

Berganza et al. reported that the 3D GCS was associated with the indexed right ventricular EDV in patients with repaired tetralogy of Fallot [12]. We found a strong correlation between 3D GCS and indexed EDV, indexed ESV, EF in the Fontan repair patients. Meanwhile, the 3D strain showed better reproducibility than the 2D strain in our study. 3D GCS showed the best inter-observer agreement (ICC = 0.87, CoV = 5.15), followed by 3D GLS (ICC = 0.84, CoV = 5.36) as compared to 2D GCS (ICC 0.82, CoV = 5.73). Previous studies reported that CMR-FT–derived 2D GLS had the best intermodality agreement among the indices measured, followed by 2D GCS, whereas 2D GRS showed more degrees of divergence between CMR-FT and speckle tracking echocardiography [26, 27]. Our results consistently indicated the high clinical applicability of 3D GCS and GLS as most robust parameters. In our study, the coefficient of variation of 3D GRS was not good (Fig. 4). GRS represented strain throughout the entire myocardial wall from subepicardium to subendocardium and was consequently much more affected by through plane motion and complex diastolic and systolic twisting motion [28].

Our study was limited by its single-center design. The retrospective nature of the study also limited its population to those clinically referred for CMR, allowing for a potential selection bias. Due to the limitations of acquisition time, we collected SSFP cine images in the four-chamber, two-chamber, and short-axis views, and the three-chamber view was not included. Result had been affected on the 2D and 3D strain analysis without three-chamber view, this is also the limitation of retrospective study. There was evidence from previous studies that the problem of twisting motion and out of plane motion could affected using 3D strain compared with 2D algorithms. In addition, 3D images presented a substantially lower spatial and temporal resolution than their 2D counterpart. We inferred that these findings might be the reason why 3D GRS results are less stable and effective [29]. Furthermore, strain rate analysis was not included in our research because the temporal resolution of the CMR cine images was somewhat lower than what is commonly recommended for strain rate evaluation in children [24]. Previous literature reported that 3D GLS in healthy subjects had a remarkably lower values compared with 2D GLS, reference values of GLS using 2D speckle tracking analysis and 3D speckle tracking analysis was 21 and 19%, respectively. The current 3D FT software has a significant drawback to measure 3D GLS [30]. Further studies are needed to assess Fontan repair patients using 3D strain and 3D speckle-tracking echocardiography.

## Conclusion

3D GCS is feasible, highly reproducible, and strongly correlated with conventional cardiac function measures. 3D GCS assessments may be useful for monitoring abnormal myocardial motion in patients with Fontan circulation.

## Data Availability

The datasets used and/or analysed during the current study are available from the corresponding author on reasonable request.

## Abbreviations

2D: Two-dimensional
3D: Three-dimensional
BSA: Body surface area
B-SSFP: Balanced steady-state free precession
CHD: Congenital heart disease
CMR: Cardiac magnetic resonance
CoV: Coefficient of variation
DENSE: Displacement encoding with stimulated echoes
ECHO: Echocardiography
EDV: End-diastolic volume
EF: Ejection fraction
ESV: End-systolic volume
FSV: Functional single ventricle
FT: Feature tracking
GCS: Global circumferential strain
GLS: Global longitudinal strain
GRS: Global radial strain
ICC: Intraclass correlation coefficient
SV: Stroke volume
